# Identification of immune activation-related gene signature for predicting prognosis and immunotherapy efficacy in lung adenocarcinoma

**DOI:** 10.3389/fimmu.2023.1217590

**Published:** 2023-07-07

**Authors:** Weibiao Zeng, Jin Wang, Jian Yang, Zhike Chen, Yuan Cui, Qifan Li, Gaomeng Luo, Hao Ding, Sheng Ju, Baisong Li, Jun Chen, Yufeng Xie, Xin Tong, Mi Liu, Jun Zhao

**Affiliations:** ^1^ Institute of Thoracic Surgery, The First Affiliated Hospital of Soochow University, Suzhou, China; ^2^ Department of Thoracic Surgery, The First Affiliated Hospital of Soochow University, Suzhou, China; ^3^ Department of Pharmaceutics, College of Pharmaceutical Sciences, Soochow University, Suzhou, China

**Keywords:** lung adenocarcinoma, immune activation, immune infiltration, immunotherapy efficacy, prognosis

## Abstract

**Background:**

Lung adenocarcinoma (LUAD) is a major subtype of non-small cell lung cancer (NSCLC) with a highly heterogeneous tumor microenvironment. Immune checkpoint inhibitors (ICIs) are more effective in tumors with a pre-activated immune status. However, the potential of the immune activation-associated gene (IAG) signature for prognosis prediction and immunotherapy response assessment in LUAD has not been established. Therefore, it is critical to explore such gene signatures.

**Methods:**

RNA sequencing profiles and corresponding clinical parameters of LUAD were extracted from the TCGA and GEO databases. Unsupervised consistency clustering analysis based on immune activation-related genes was performed on the enrolled samples. Subsequently, prognostic models based on genes associated with prognosis were built using the last absolute shrinkage and selection operator (LASSO) method and univariate Cox regression. The expression levels of four immune activation related gene index (IARGI) related genes were validated in 12 pairs of LUAD tumor and normal tissue samples using qPCR. Using the ESTIMATE, TIMER, and ssGSEA algorithms, immune cell infiltration analysis was carried out for different groups, and the tumor immune dysfunction and rejection (TIDE) score was used to evaluate the effectiveness of immunotherapy.

**Results:**

Based on the expression patterns of IAGs, the TCGA LUAD cohort was classified into two clusters, with those in the IAG-high pattern demonstrating significantly better survival outcomes and immune cell infiltration compared to those in the IAG-low pattern. Then, we developed an IARGI model that effectively stratified patients into different risk groups, revealing differences in prognosis, mutation profiles, and immune cell infiltration within the tumor microenvironment between the high and low-risk groups. Notably, significant disparities in TIDE score between the two groups suggest that the low-risk group may exhibit better responses to ICIs therapy. The IARGI risk model was validated across multiple datasets and demonstrated exceptional performance in predicting overall survival in LUAD, and an IARGI-integrated nomogram was established as a quantitative tool for clinical practice.

**Conclusion:**

The IARGI can serve as valuable biomarkers for evaluating the tumor microenvironment and predicting the prognosis of LUAD patients. Furthermore, these genes probably provide valuable guidance for establishing effective immunotherapy regimens for LUAD patients.

## 1 Introduction

Lung cancer is a highly prevalent and lethal malignancy on a global scale (1). Common histologic subtypes of lung cancer encompass lung squamous cell carcinoma (LUSC), lung adenocarcinoma (LUAD), and small cell lung cancer (SCLC), with LUAD representing the most predominant subtype (2). Current therapeutic approaches for LUAD include surgery, chemotherapy, radiotherapy, targeted therapies, and Immunotherapy; However, these modalities are only efficacious in a subset of LUAD patients, and the prognosis for those with advanced LUAD remains unfavorable (3). Notwithstanding clinical parameters, such as the tumor-node-metastasis (TNM) staging system, vascular invasion, and tumor mutation load status, have been extensively utilized, these prognostic and predictive factors have limitations in their capacity to accurately forecast the prognosis and therapeutic response of LUAD patients (4, 5). Therefore, novel prognostic classifiers or therapeutic biomarkers are urgently needed to enhance the clinical benefit for patients with LUAD.

Emerging evidence suggests that the tumor immune microenvironment (TIME) plays a pivotal role in the initiation and progression of neoplasms (6). As a complex and heterogeneous ecosystem, the TIME, encompassing various stromal, vascular, and immune cells, has been identified as a putative determinant of cancer therapeutic response (7, 8). Immune checkpoint inhibitors (ICIs), one of the most promising immunotherapeutic strategies can enhance the anti-tumor properties of effector T cells by ameliorating their dysfunction and depletion promoting their activation and function (9). Consequently, they have been demonstrated to extend patient survival across a spectrum of malignancies, including melanoma, breast, liver, and urothelial carcinoma (10, 11). Several ICIs have been approved for cancer therapy, such as Nivolumab and Keytruda, which target Programmed Death Receptor1 (PD-1), Atezolizumab, which targets Programmed cell death 1 ligand 1 (PDL1), and Ipilimumab, which targets cytotoxic T-lymphocyte-associated protein 4 (CTLA-4) (12–14). Unfortunately, ICIs do not always succeed in restoring suppressed T cells repertoire in cancer patients, and some ICI users experience serious immunological adverse effects (15). Recent studies have demonstrated that hot tumors, also known as tumors with a high number of immune infiltrating cells in a pre-activated state, respond well to ICIs (16–19). Whereas cold tumors that lack infiltrating immune cells show little immune system activation (20). In advanced LUAD patients, the TIME is characterized by a high abundance of immunosuppressive cells and stromal components that create a hostile milieu for T cell function by secreting various metabolites, chemokines, and cytokines. As a result, this group of LUAD patients exhibits poor immune activation and response to ICIs (20). Hence, it is crucial to evaluate the immune activation status of the TIME for personalized therapy in LUAD patients.

The immune activation signature mainly includes the IFNγ signature, the expanded immune gene signature, the cytotoxic T lymphocyte (CTL) signature, and the expression of MHC class I molecules HLA-A and HLA-B, All these signatures have been previously reported to correlate with the prognosis of solid tumors (20–23). In this study, we constructed an immune activation related gene index (IARGI) risk model that uses identified prognostic genes associated with LUAD survival to predict prognosis and immunotherapy outcomes in patients with LUAD.

## 2 Materials and methods

### 2.1 RNA extraction and quantitative real time-PCR

Tumour tissues and corresponding normal tissues were collected from 12 LUAD patients from the First Affiliated Hospital of Soochow University (Suzhou, China). The clinical information of the patients is provided in **Table S1** of **Supplementary Materials**. Tumour staging was performed according to the Union for International Cancer Control Tumour Node Metastasis (TNM) classification of lung carcinoma (24). Total RNA was extracted from fresh tissue samples using Trizol, followed by reverse transcription to cDNA and quantitative real-time polymerase chain reaction (qPCR) analysis. The qPCR data was normalized to β-actin using the 2-ΔΔCt method. The primer sequences used for qPCR are listed in **Table S2** of **Supplementary Materials**. This study was approved by the Ethics Committee of First Affiliated Hospital of Soochow University (20228181).

### 2.2 Data collection of databases

The TCGA database (The Cancer Genome Atlas) was used to retrieve RNA expression profiles, clinical data, genetic mutations, and copy number variation (CNV) data for LUAD in the training set. Finally, after data cleaning, 495 samples were collected from TCGA for further analysis. Using the Gene Expression Omnibus (GEO) database, we obtained the RNA expression profiles and clinical data for the GSE72094 dataset. PRJEB23709 and GSE135222 were two datasets collecting cancer patients treated with immune checkpoint inhibitors, downloaded from the European Nucleotide Archive and GEO, respectively. All the obtained RNA expression profiles were log Log2(TPM +1) transformed for normalization. IAGs were identified from three previously published gene sets, namely the IFN gamma signature (22), the expanded immune gene signature (25), the cytotoxic T lymphocyte (CTL) signature (26), and the prognosis-related HLA-A and HLA-B genes (27). Protein-protein interaction study were conducted by an open-source STRING database (https://cn.string-db.org).

### 2.3 Consensus clustering

By Using the R package “ConsensusClusterPlus” (28), an unsupervised consensus clustering analysis was carried out to investigate the expression profile data of the prognostic genes. The optimal number of clusters was selected based on the cumulative distribution curve and the process was repeated 1000 times to ensure the stability of the results.

### 2.4 Tumor immune microenvironment

Multiple algorithms, including ESTIMATE (29), TIMER (30) and the single sample gene set enrichment analysis (ssGSEA) algorithm (31) were applied to analyze the tumor microenvironment. The ESTIMATE algorithm evaluates the ESTIMATE score, immune score, and stromal score, the TIMER algorithm and the ssGSEA algorithm were utilized to estimate the infiltration abundance of various types of immune cells.

### 2.5 Prediction of immunotherapy response

The Tumor Immune Dysfunction and Rejection (TIDE) score is a web-based tool (HTTP://tide.dfci.harvard.edu/) that evaluates the potential clinical efficacy of immunotherapy in different risk groups and indicates the potential for tumor immune evasion (21); a higher TIDE score implies a poorer response to ICI. SIGLEC15, TIGIT, CD274, HAVCR2, PDCD1, CTLA4, LAG3, and PDCD1LG2 are genes related to immune checkpoints (32). We extracted the expression values of these eight genes to examine the expression of immune checkpoint-related genes in different groups.

### 2.6 Functional analyses

Differential genes between the two IAG patterns were obtained using the R package “limma” (33). To compare the differential pathways and biological effects between the two genetic subtypes, we performed Kyoto Encyclopedia of Genes and Genomes (KEGG) analysis with Cluster Profiler R package (version 3.14.3) and Gene Ontology (GO) analysis with Metascape website based on DEGs (|FC| >1.5, P < 0.05) (34, 35). The KEGG analysis was performed using the R package “Cluster Profiler” (version 3.14.3) and the GO analysis was performed on the Metascape website (36). We also obtained immune activation-related gene/protein interactions from the STRING website (http://www.string-db.org/) and constructed the network using Cytoscape software. We calculated Degree scores and screened core genes (Degrees > 10) with Cytoscape software (37).

### 2.7 Construction of the IAG-related risk signature

We performed LASSO-Cox analysis (10-fold cross-validation) with glmnet R package using IAGs that were statistically significant in univariate Cox regression analyses. To minimize the risk of overfitting, we performed 100 LASSOs and selected genes that appeared multiple times in the model. Ultimately, a linear equation called “IARGI” was constructed to predict the overall survival of LUAD patients: risk score = [coef (1) × GeneExp (1)] + [coef (2) × GeneExp (2)] +… + [coef(i) × GeneExp(i)] (38). We used Kaplan-Meier curves with survival and survminer R packages to perform prognostic analyses and assess survival at 1, 3, and 5 years.

### 2.8 Tumor immune single cell hub database

Expression analysis of immune-activation-related prognostic genes at single-cell resolution was conducted using data from the NSCLC-EMTAB6149 dataset (39) through the Tumor Immune Single Cell Hub Database (http://tisch.comp-genomics.org/home/) (40), an online database focused on the tumor microenvironment (TME) that has collected single-cell transcriptome profiles of nearly 20,000 cells from 27 tumor datasets across 76 cancers. The NSCLC-EMTAB6149 dataset contains a 52,698-cell catalog of the TME transcriptome in human lung tumors at single-cell resolution.

### 2.9 Prediction of chemotherapeutic drug sensitivity

The R package “pRRophetic” was used to predict the sensitivity of chemotherapeutic drugs (41). The minimum inhibitory concentration (IC_50_) was calculated based on the expression profiles of different risk groups of LUAD patients, and the specific drug for the group was selected by comparing the sensitivity of chemotherapeutic drugs in the high and low-risk groups.

### 2.10 Statistical analysis

Statistical analyses were performed with R (version 3.6.1) and GraphPad Prism (version 8.0.1). Survival analyses were performed using the Kaplan-Meier method, and the predictive performance of the risk model was assessed by the “survivalROC” R package using time-dependent subject work characteristics (ROC). Discontinuous data were expressed as numbers/percentages, and continuous data were expressed as mean ± standard deviation (SD). Statistical analysis was performed using Student’s t-test between the two groups. p < 0.05 was considered a statistically significant difference.

## 3 Results

### 3.1 Transcriptional and genetic alterations of immune activation genes in LUAD patients

**Figure 1:**
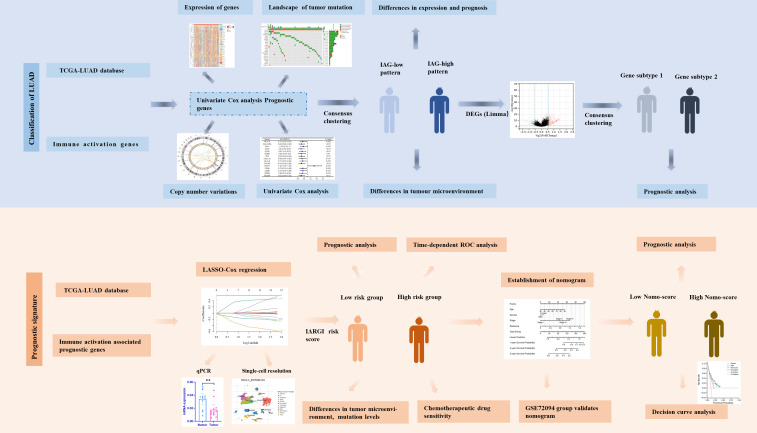
Flow chart of the data analyzing process.

**Figure 2:**
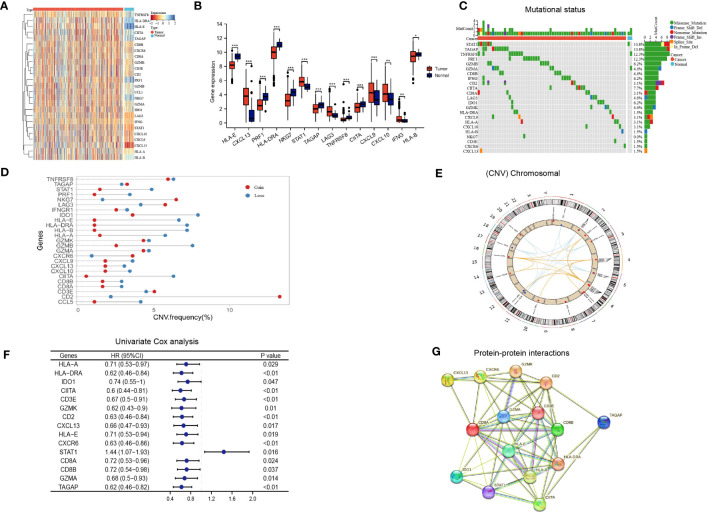
Immune activation-related genes in the TCGA LUAD cohort. **(A, B)** Differential expression of immune activation-related genes between tumor and normal tissues presented in a heatmap **(A)** and the box plot **(B)**, *P < 0.05, **P < 0.01, ***P < 0.001; **(C)** The mutation landscape of immune activation-related genes; **(D, E)** Copy number variations of immune activation-related genes; **(F)** Univariate Cox analysis identifying prognostic genes; **(G)** Protein-protein interactions among immune activation associated prognostic genes.

The flow chart of our study is illustrated in **Figure 1**. We integrated a set of 25 IAGs reported in the literature (**Supplementary Table S3**), and compared their expression patterns between normal and tumour tissues in the TCGA LUAD cohort. Eight IAGs, namely HLA-E, PRF1, HLA-DRA, NKG7, HLA-B, TAGAP, TNFRSF8, and CIITA were found to be significantly downregulated in LUAD, while six genes, namely IFNG, CXCL13, LAG3, SATA1, CXCL9 and CXCL10 were significantly upregulated (**Figures 2A, B**). To further investigate the value of the 25 IAGs in LUAD, we analyzed their mutational status in the TCGA LUAD cohort (**Figure 2C**). In the included LUAD tumour tissues, mutations in these genes were detected in 74 cases at a frequency of 14.9%. Among these genes, STAT1 had the highest mutation frequency, followed by TAGAP, TNFRSF8, PRF1, GZMB, and GZMA. Conversely, mutations in these genes were rare in normal tissues. Copy number variation (CNV) analysis indicated that copy number gain was prevalent in CD2, NKG7, and TNFRSF8, while IDO1 and GZMB were primarily associated with copy number loss (**Figure 2D**). CNV was observed on multiple chromosomes, particularly on chromosomes 2, 4, 5, 6, 11, and 12 (**Figure 2E**). Overall, our analysis revealed a significant degree of heterogeneity in the genetic and transcriptional changes of IAGs between tumour and normal specimens, suggesting their crucial role in tumour initiation and progression.

Based on the aforementioned IAGs, 15 genes that are potentially associated with LUAD prognosis were screened by Kaplan Meier survival analysis and univariate Cox analysis using Kaplan-Meier Plotter (http://kmplot.com/analysis/) (**Figure 2F**). Results from protein-protein interaction (PPI) analysis revealed the presence of interactions between these genes/proteins, which were validated through experimental assays (pink lines), curated databases (blue lines), co-expression (black lines), or text mining (olive green lines) (**Figure 2G**).

### 3.2 Identification of two different molecular patterns of LUAD based on IAGs

**Figure 3:**
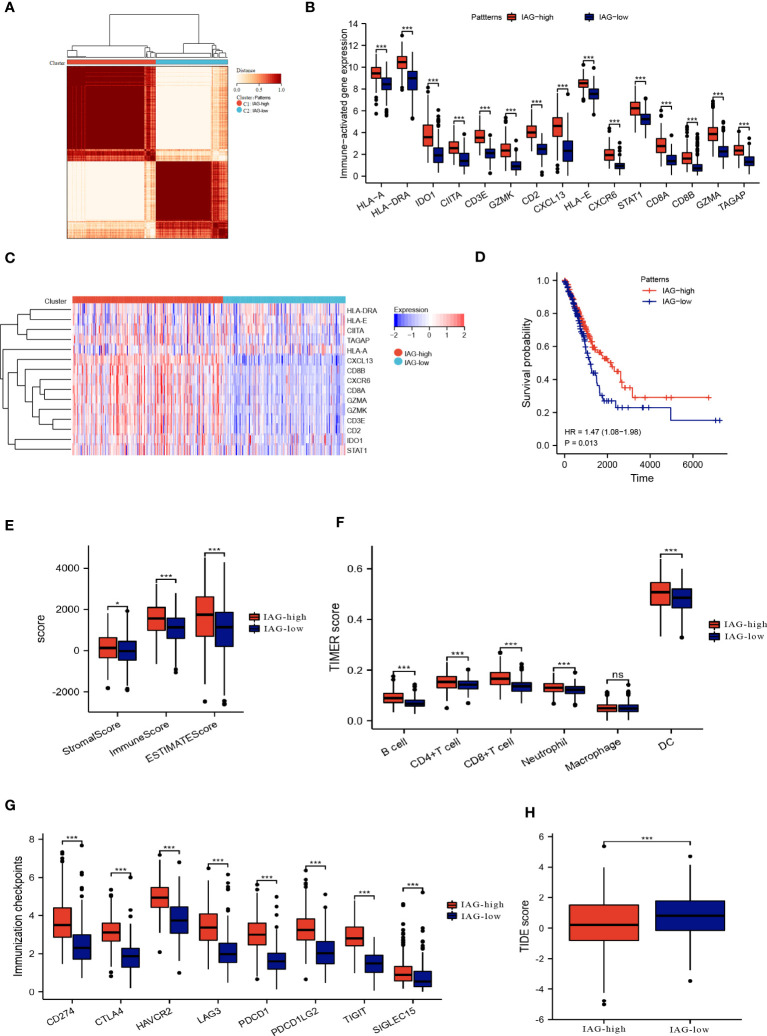
Two patterns based on immune activation associated prognostic genes revealed differences in the tumor microenvironment. **(A)** Consensus clustering of 15 prognostic genes in TCGA LUAD cohort; **(B, C)** The expression of immune activation associated prognostic genes between the two patterns; **(D)** Differences in prognosis between the two patterns; **(E)** ESTIMATE score, immune score and the stromal score of the two patterns; **(F)** Abundance of infiltration of immune cells calculated by TIMER algorithm; **(G)** The expression of immune checkpoint-associated genes in the IAG-high and IAG-low patterns; **(H)** TIDE score of the two patterns. *P<0.05, ***P < 0.001. The expanded form of "ns" represents "not significant". This indicates that the statistical analysis did not yield any meaningful results.

By employing consensus clustering methods, two distinct clusters were discerned in the TCGA LUAD cohort based on differential expression patterns of IAGs (**Figure 3A**). Cluster 1 had high expression levels of these genes and was defined as the IAG-high pattern. Cluster 2 had low expression levels and was defined as the IAG-low pattern (**Figures 3B, C**). Notably, survival analysis revealed a significant survival discrepancy between the two patterns, with a more favorable prognosis associated with the IAG-high pattern and a poorer prognosis linked to the IAG-low pattern (**Figure 3D**).

### 3.3 Tumor microenvironment landscape in two molecular patterns

The ESTIMATE algorithm was used to compare the immune microenvironments of the two models, and the findings revealed that the IAG-high model had higher ESTIMATE, immunological, and stromal scores (**Figure 3E**). According to the TIMER scores, the IAG-low pattern was associated with a decreased abundance of immune cells, including B cells, CD4^+^T cells, CD8^+^ T cells, and dendritic cells, as evidenced by **Figure 3F**. Moreover, a comparative analysis of immune checkpoint-associated genes in both molecular patterns revealed a significantly higher expression of these genes in the IAG-high pattern as compared to the IAG-low pattern (**Figure 3G**).

The Tumor Immune Dysfunction and Exclusion (TIDE) model is a computational tool that simulates two primary mechanisms of tumor immune evasion and predicts the efficacy of immunotherapy (42). Specifically, a high TIDE score indicates the presence of suppressor cells that may hinder T-cell infiltration. In this study, the TIDE score was calculated for both IAG molecular patterns, and the results revealed a significantly lower TIDE score in the IAG-high pattern compared to the IAG-low pattern, indicating that patients with the former molecular pattern may derive more benefits from immunotherapy (**Figure 3H**).

### 3.4 Construction of genomic subtypes based on differentially expressed genes from two IAG patterns

**Figure 4:**
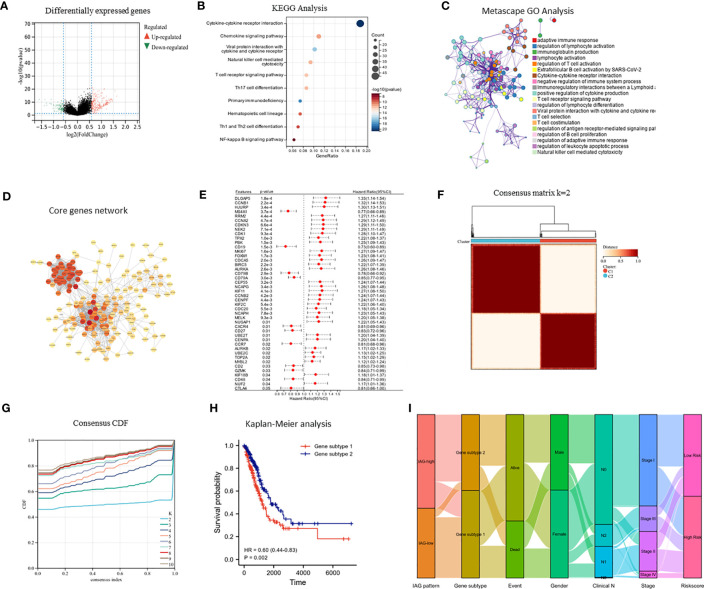
Identification of immune activation related gene subtypes based on differentially expressed genes. **(A)** Volcano map showed differentially expressed genes between two patterns (|FC| >1.5, P < 0.05); **(B)** KEGG enrichment analysis of differentially expressed genes; **(C)** GO enrichment analysis of differentially expressed genes using Metascape; **(D)** Construction of core gene network using STRING database and cytoscope software; **(E)** Univariate cox analysis of core genes; **(F, G)** Consensus clustering based on core genes; **(H)** Prognostic differences between two genomic subtypes. **(I)** Sankey diagram based on IAG patterns, gene subtypes, gender, pathological stage, survival status and risk score.

The differential expression analysis between the two IAG patterns was conducted using the “limma” R package, with a threshold of |FC| >1.5 and p< 0.05. A total of 377 DEGs were identified, comprising 276 upregulated genes in the IAG-high pattern and 101 upregulated genes in the IAG-low pattern (**Figure 4A**). The KEGG pathway analysis based on these DEGs revealed significant enrichment of immune-related biological processes, such as cytokine-cytokine receptor interaction, T cell receptor signaling pathway, chemokine signaling pathway, and natural killer cell-mediated cytotoxicity (**Figure 4B**). Moreover, GO analysis was performed using a metascape, and the enriched modules were displayed in different colored regions (**Figure 4C**). In these modules, several biological functions were consistent with the KEGG analysis, such as regulation of lymphocyte activation in adaptive immune response, immunoglobulin production, lymphocyte activation, and regulation of T cell activation. To further investigate the crucial genes that play a central role in these differentially expressed genes, a core PPI network was constructed using the STRING database (network type: physical subnetwork, minimum required interaction score: 0.4) and Cytoscape software (Cytohubba plugin, Degree > 15). Ultimately, a core network containing 68 genes was obtained (**Figure 4D**). Univariate Cox analysis of 68 core genes revealed that 46 of them were associated with prognosis (**Figure 4E**). Using an unsupervised consensus clustering approach based on the expression of 46 core genes, we classified LUAD patients from the TCGA cohort into two genomic subtypes: gene subtype 1 and gene subtype 2 (**Figures 4F, G**). Gene subtype 1 had a significantly worse prognosis than gene subtype 2 (**Figure 4H**). To better understand the direct relationship between IAG patterns and gene subtypes, Sankey plots were drawn and it was observed that the majority of IAG high pattern and a small proportion of IAG low pattern comprise gene subtype 1 (**Figure 4I**).

### 3.5 Construction of immune activation-related gene index risk model

**Figure 5:**
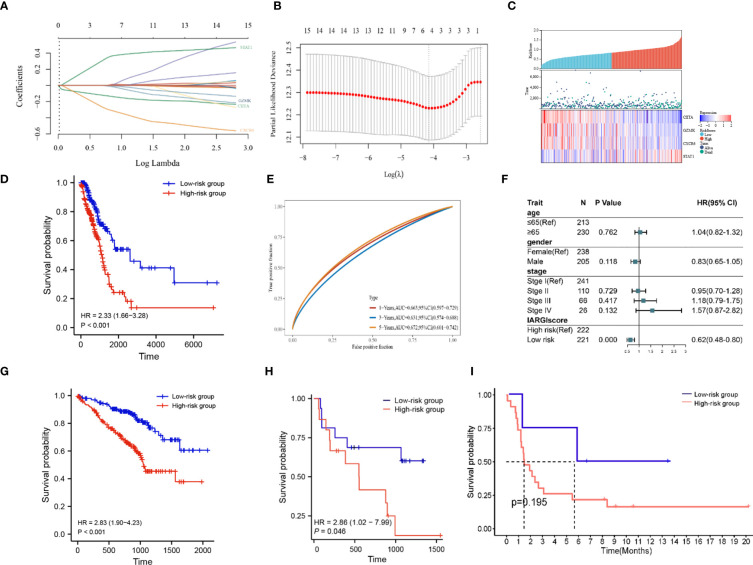
Construction of prognostic signature by IARGI. **(A, B)** LASSO-Cox regression analysis based on 15 prognostic genes; **(C)** Differences in survival status and expression of 4 IARGI genes between different risk groups; **(D)** Prognostic analysis of two risk groups from TCGA LUAD cohort; **(E)** Time-dependent ROC analysis of TCGA LUAD cohort; **(F)** Multivariate Cox analysis of IARGI risk score and clinical factors; **(G)** Prognostic analysis of two risk groups from GSE72094; **(H)** Prognostic analysis of two risk groups from PRJEB23709; **(I)** Prognostic analysis of two risk groups from PRJEB23709.

Based on LASSO-Cox regression analysis results (**Figures 5A, B**), four IAGs were selected as the best indicators for the prediction model. The risk score was computed as follows: risk score = (-0.1283) * (CIITA expression) + (-0.0075) * (GZMK expression) + (-0.2923) * (CXCR6 expression) + (0.3394) * (STAT1 expression), these results were then incorporated into the IARGI risk model. Firstly, we utilized the expression levels and risk coefficient of 4 IARGI genes in the LUAD dataset to calculate the IARGI risk scores of each patient. Based on the median of the risk scores, LUAD patients were categorized into low-risk and high-risk groups, respectively. The heat map showed the expression of 4 genes in both groups and revealed significantly higher mortality in the high-risk group (**Figure 5C**). The overall survival rate of patients in the low-risk group was significantly better than that of the high-risk group (**Figure 5D**). Time-dependent ROC analysis demonstrated that our IARGI risk model had good predictive power over a 5-year time horizon (**Figure 5E**). We performed multivariate Cox analysis on LUAD patients by IARGI, age, gender, and pathological stage, the results suggested that IARGI could be an independent prognostic factor (**Figure 5F**). IARGI also showed good performance in predicting the prognosis of LUAD samples in the validation set (GSE72094) (**Figure 5G**). We explored the ability of the IARGI risk score to assess the effect of immunotherapy in two cohorts of cancer patients treated with immune checkpoint inhibitors, PRJEB23709 and GSE135222. The results of KM survival analysis showed that the IARGI risk score was associated with longer overall survival after treatment with ICIs (**Figure 5H**). Analysis of data from GSE135222 also found a potential difference in progression-free survival for patients with different IARGI scores treated with ICIs (**Figure 5I**), although this difference was not statistically significant due to the small sample size and the short follow-up period.

To further elucidate the correlation between IARGI and clinical factors, we conducted a comprehensive analysis. Our results demonstrate that there was no statistical significance observed in the IARGI risk scores of patients with an age greater than or equal to 65 years and those under 65 years (**Supplementary Figure S1A**). In contrast, the male gender was associated with significantly higher risk scores compared to the female gender (**Supplementary Figure S1B**). Additionally, patients with pathological stages III and IV exhibited higher risk scores than those with pathological stages I and II (**Supplementary Figure S1C**).

### 3.6 Expression validation and single-cell resolution of 4 IARGI genes

**Figure 6:**
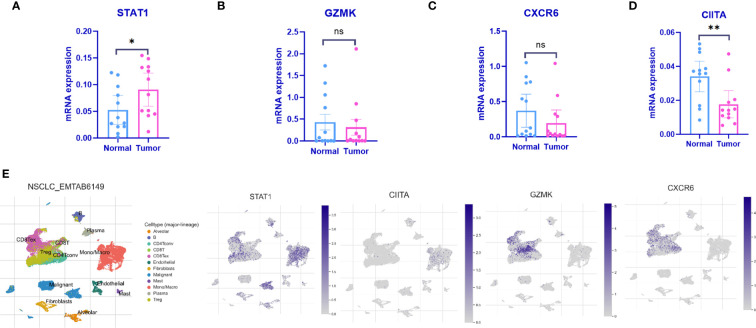
Expression of the 4 IARGI genes in LUAD verified by qPCR and Single-cell dataset **(A–D)** The expression of 4 IARGI genes in 12 pairs LUAD tumors and normal tissues was verified by qPCR; **(E)** The distribution of 4 IARGI genes in different cell types was analyzed using single-cell resolution in the NSCLC-EMTAB6149 dataset by UMAP. Mean ± standard deviation. *P<0.05, **P<0.01. The expanded form of "ns" represents "not significant". This indicates that the statistical analysis did not yield any meaningful results.

qPCR was employed to validate the expression levels of the 4 IARGI genes in LUAD tumor tissues and normal tissues. Clinic information could be found in **Table S1**. The results indicated a significant upregulation of STAT1 expression in tumor tissues compared to normal tissues, while the expression of CIITA showed the opposite trend (**Figures 6A–D**), which is consistent with the results of previous bioinformatics analysis. The NSCLC-EMTAB6149 dataset was analyzed at the single-cell resolution using UMAP dimensionality reduction method to investigate the distribution patterns of four IARGI genes across different cell types (39). It was discovered that CIITA, GZMK, and CXCR6 are predominantly expressed in T cells and monocytes/macrophages, while STAT1 exhibits high expression in both malignant and immune cells (**Figure 6E**).

### 3.7 Predicting immune infiltration, genetic mutations, and chemotherapeutic drug efficacy based on IARGI

**Figure 7:**
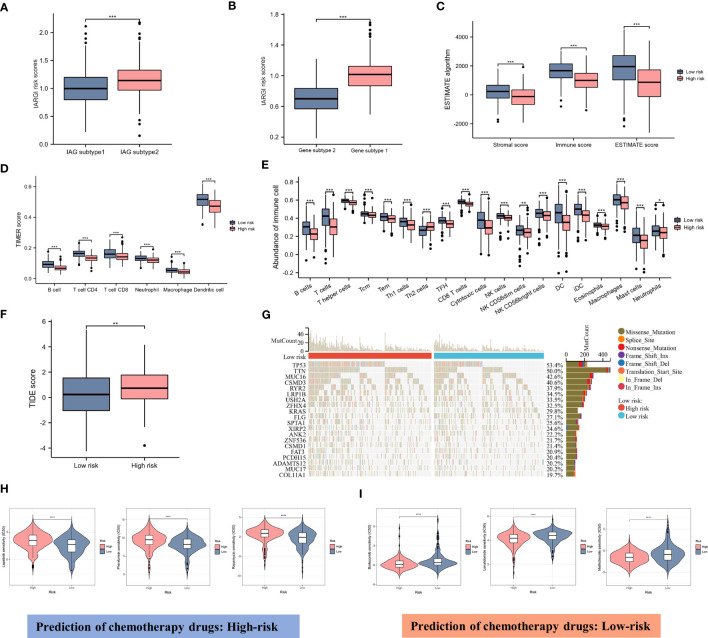
Correlation between IARGI and TME, mutation levels, chemotherapeutic drug sensitivity. **(A)** IARGI risk scores in IAG-high and IAG-low patterns; **(B)** IARGI risk scores in the two different gene subtypes; **(C)** Stromal score, immune score, and estimate score between high- and low-risk groups; **(D)** Evaluation of immune cell infiltration in high-risk and low-risk groups by TIMER algorithm; **(E)** Evaluation of immune cell infiltration in high-risk and low-risk groups by ssGSEA algorithm. **(F)** TIDE score in high-risk and low-risk groups; **(G)** Genetic mutation in high-risk and low-risk groups; **(H, I)** Prediction of chemotherapeutic drugs in high-risk and low-risk groups. *P<0.05, **P<0.01, ***P < 0.001.

As previously described, two molecular patterns and two genetic subtypes associated with IAG risk signature were identified. As shown in **Figures 7A, B**, the IAG-low pattern and gene subtype 1 had higher IARGI risk scores, and these findings were consistent with previous analyses. Following the validation of the prognostic utility of the IARGI, we sought to investigate the potential biological significance of the IARGI genes in the context of tumour immune response. Specifically, we aimed to determine whether IARGI risk score could be utilized for predicting immune infiltration, gene mutation status, and immunotherapy outcome prediction in patients with LUAD. We utilized the ESTIMATE algorithm, which revealed that individuals in the high-risk group exhibited lower estimated, immune, and interstitial scores, indicating a lower level of immune infiltration (**Figure 7C**). These findings highlight the potential clinical utility of the IARGI as a predictive biomarker for immunotherapy response in LUAD patients. Further research is warranted to elucidate the underlying molecular mechanisms involved in this process. The immune cells associated with antitumor immunity were more abundant in the low-risk group than in the high-risk group, including CD4^+^ T cells, CD8^+^ T cells, DC, and macrophage, except for Th2 cells associated with immunosuppression. These results suggested that the high-risk group had a negative TME that promotes tumours progression (**Figures 7D, E**). In addition, our study found that the IARGI risk score was significantly negatively correlated with PDL1 expression and positively correlated with tumours mutational load (TMB) (**Supplementary Figure S2**).

TIDE score was used to evaluate the prognostic value of the IARGI risk score in the context of immunotherapy efficacy. The results of TIDE demonstrated that the low-risk cohort exhibited a significantly lower TIDE score (**Figure 7F**), suggesting that patients with a lower IARGI risk score may derive greater therapeutic benefits from immunotherapy. The heat map revealed the 20 genes with the highest mutation rates in both groups and demonstrated a significant difference in the mutation frequency of these genes. The low-risk group had mutations in 86.8% (190/219) of samples, whereas the high-risk group `had mutations in 96.4% (214/222) of samples (**Figure 7G**). We also performed chemotherapeutic drug predictions for patients with LUAD in the high- and low-risk groups to provide personalized treatment options. Lapatinib, Rapamycin, and Phenformin were more effective for the high-risk group, while Bortezomib, Lenalidomide, and Methotrexate were more effective for the low-risk group (**Figures 7H, I**).

### 3.8 Construction and calibration of a nomogram that combines clinical factors with IARGI

**Figure 8:**
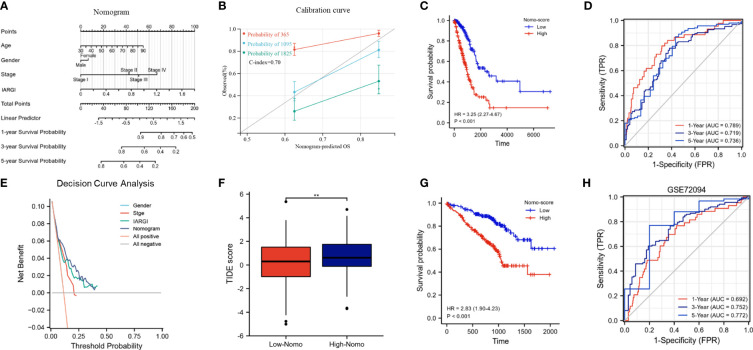
Construction and calibration of the nomogram. **(A)** Nomogram integrating IARGI and clinical features; **(B)** Calibration curve of 1-year, 3-year, and 5-year survival time. **(C)** Kaplan-Meier analyses of the training set (based on nomo-score); **(D)** Time-dependent ROC curves of the training set (based on Nomo-score); **(E)** Decision curve analysis curve of nomogram score, IARGI, gender, and pathological stage; **(F)** Correlation between Nomo-score and TIDE score; **(G)** Kaplan-Meier analyses of the validation set (based on Nomo-score); **(H)** Time-dependent ROC curves of the validation set (based on Nomo-score). **P<0.01.

We constructed a nomogram by combining the IARGI with clinical factors to improve the accuracy of prognosis prediction (**Figure 8A**). The calibration curve showed good agreement between the nomogram-predicted and observed survival times at 1, 3, and 5 years (**Figure 8B**). The survival curves and time-dependent ROC curves also indicated that the nomogram had a better prognostic value than the IARGI alone (**Figures 8C, D**). The decision curve analysis (DCA) revealed that using the nomogram score or the IARGI risk score for survival prediction was more beneficial than using the pathological stage alone (**Figure 8E**). Results of the TIDE algorithm show that the Nomo score is a good predictor of immunotherapy outcome (**Figure 8F**). We validated the nomogram using an external validation set (GSE72094). The results showed that the Nomo score significantly stratified LUAD patients into different prognostic groups: patients with higher Nomo scores had worse survival outcomes (**Figure 8G**). The time-dependent ROC curve also confirmed the validity of the nomogram (**Figure 8H**). These results further demonstrated that our nomogram model, which integrated IARGI and clinical factors, improved the accuracy of prognosis prediction in LUAD patients.

## 4 Discussion

Pre-activated immune status of the tumours microenvironment is widely considered a powerful predictor of response to cancer therapy and survival outcome (43). In this study, we developed a novel immune-related predictive model utilizing immune-activating genes and multiple statistical approaches to accurately predict the prognosis and immunotherapy response of LUAD. Using both the training dataset (TCGA-LUAD) and an external validation dataset (GSE72094), we stratified LUAD patients into high-risk and low-risk groups based on IARGI, with high-risk patients exhibiting poorer prognosis and immunotherapy response, as expected. Furthermore, we established a nomogram model incorporating IARGI and clinical features in predicting patient overall survival in LUAD, thereby significantly enhancing prognostic accuracy and holding immense potential for clinical application. Our findings demonstrate the reliability of IARGI as robust biomarkers for predicting the prognosis and immunotherapy response of LUAD and provide valuable insights for personalized treatment strategies in LUAD patients.

The tumor immune microenvironment is closely related to tumor occurrence, treatment response, and drug resistance (44, 45). An increasing number of immune-related gene signatures have been used for prognostic classification of LUAD (46, 47), however, limited studies have been performed to characterize immune activation-related genes in the immune microenvironment of LUAD. One important aspect to consider is that certain IAGs are expressed in both tumor and immune cells. As both RNA sequencing data from TCGA and mRNA expression levels detected by qPCR represent the cumulative expression of all cell types in a given tissue, it may be difficult to distinguish the source of these genes. Consequently, the RNA expression levels of these IAGs may not show significant differences between tumor and normal tissues. In this study, 15 immune-associated prognostic genes in LUAD were identified and their mutation frequency and CNV were analyzed. Based on the expression of prognostic genes, LUAD patients were classified into two patterns, viz. IAG-high pattern and IAG-low pattern. Patients in IAG-high pattern have longer survival and more abundant immune cells such as B cells, DCs, neutrophils, macrophages, CD4^+^ T cells, and CD8^+^ T cells. This indicates that high expression of IAGs is closely related to lymphocyte infiltration in tumors. Moreover, previous studies have shown that high-density tumor-infiltrating lymphocytes can inhibit tumor progression (48), especially in patients who receive immune checkpoint inhibitors (49). TIDE scores can predict patient response to immunotherapy by assessing the potential ability of tumor immune evasion. In our results, patients in the IAG-high pattern had significantly lower TIDE scores than in the IAG-low pattern, which also suggested that patients with the IAG-high pattern benefited more from ICI treatment than those with the IAG-low pattern.

To stratify the risk of patients with LUAD accurately, we constructed a prognostic signature based on four genes associated with immune activation: CIITA, GZMK, CXCR6, and STAT1. CIITA is the main transcriptional activator of MHC class II molecules. It changes the closed chromatin and promoter of inactive MHC II genes to an open conformation, facilitating the binding of trans-acting factors to the promoter (50). Low expression of CIITA can impair antigen processing and presentation by MHC class I and II molecules (51), thus weakening anti-tumor immunity and enhancing tumor growth. High expression of GZMK and CXCR6 has also been found to enhance immune surveillance and antitumor immunity in several studies (52, 53). STAT1 plays an important and complex role in tumor formation and tumor immunosurveillance: on one hand, STAT1 can exert antitumor effects by activating the interferon signaling pathway (54) and acts as a predictor of therapeutic response to adjuvant therapy in cancer patients (55). on the other hand, it plays an important role in cell migration, tissue invasion, and therapeutic resistance to radiotherapy and chemotherapy (56). STAT1 has different roles in different tumors. In LUAD, as previously reported, it mainly acts as a tumor promoter (57). Moreover, we observed that STAT1 was highly expressed in both malignant and immune cells at single-cell resolution (**Figure 6A**). Considering the importance of immune infiltration in the tumor ecosystem, we used the ESTIMATE algorithm, TIMER, and ssGSEA algorithms to analyze immune infiltration. The results suggest that populations with high IARGI exhibit lower immune infiltration, which is the main reason for their low objective response rate to immunotherapy, in agreement with previous speculations (58).

Many prognostic models for LUAD based on gene features have been developed, such as a tumor microenvironment-associated prognostic signature introduced by Zhao et al. (47), a seven-immune-related-gene signature constructed by Zhan et al (59), and an alternative splicing-related prognostic model proposed by Zhu et al. (60). Compared with these models, our study reported a prognostic model based on IARGI, which showed good prognostic accuracy for LUAD. Given that checkpoint inhibitors are currently only about 25% effective in the treatment of advanced lung cancer, and that a proportion of lung cancer patients do not have their immune systems effectively activated, prognostic models constructed based on immune activation-related genes are important for assessing the effectiveness of immunotherapy and predicting the prognosis of patients. The 4-gene prognostic model constructed in this study helps to determine prognosis, the IARGI derived from this model can serve as an independent prognostic factor and a clinically useful indicator. The combination of the IARGI and clinical factors in a nomogram can improve the accuracy and potential clinical application value. Our study has several limitations. First, the gene set in this study was based on previously published transcriptomic signatures, so some genes involved in immune activation might have been missed. Second, we acknowledge that this is a study based on public databases, and the predictive ability of our findings needs to be confirmed by independent prospective clinical studies.

## 5 Conclusions

This study aimed to construct an IARGI model using immune activation-related genes to predict the prognosis and immunotherapeutic effects of LUAD patients. The model was well validated in several aspects, and our findings provide new insights for prognostic classification and potential oncology drug discovery. Notably, IARGI can serve as a guide to clinical judgment and individualized treatment in the current era of triumphant immunotherapy in cancer treatment.

## Data availability statement

Publicly available datasets were analyzed in this study. This data can be found within the article/**Supplementary Material**.

## Ethics statement

The studies involving human participants were reviewed and approved by the Ethics Committee of First Affiliated Hospital of Soochow University (20228181). The patients/participants provided their written informed consent to participate in this study.

## Author contributions

WZ, JW, and JY extracted the data regarding the TCGA and GEO database and were major contributors in writing the manuscript. ZC, YC, and QL processed and interpreted the data. GL, HD, and SJ scrutinized the results and revised the manuscript. BL, JC, and XT wrote and reviewed the manuscript. Study supervision: ML and JZ. All authors contributed to the article and approved the submitted version.
