# Supplementary material for: Identification of immune activation-related gene signature for predicting prognosis and immunotherapy efficacy in lung adenocarcinoma
Source: Front Immunol. 2023 Jul 7;14:1217590. doi: 10.3389/fimmu.2023.1217590 (PMC10364982; doi:10.3389/fimmu.2023.1217590)
Supplement: Supplementary file 1 [file DataSheet_1.pdf]

SUPPLEMENTARY MATERIALS

Table S1. Clinical information of LUAD patients used to validate mRNA expression.

Patients	Gender	Ages	TNM stage
1	Male	47	ⅡA
2	Female	49	ⅡA
3	Male	63	ⅡA
4	Female	61	ⅡB
5	Male	53	ⅡA
6	Female	50	ⅢA
7	Male	47	ⅡA
8	Male	67	ⅡA
9	Female	69	ⅡA
10	Male	72	ⅡB
11	Male	70	ⅡB
12	Male	65	ⅡA

Table S2. The immune activation-related genes used in this study

Immune activation-related genes				
HLA-A	IFNG	GZMK	CXCR6	CD8B
HLA-B	IDO1	CD2	LAG3	GZMA
CXCL10	CIITA	CXCL13	STAT1	PRF1
CXCL9	CD3E	NKG7	GZMB	TNFRSF8
HLA-DRA	CCL5	HLA-E	CD8A	TAGAP

Table S3. Primer list used in this study

Primer names	Upstream base sequence	Downstream base sequence
CIITA	CCTGGAGCTTCTTAACAGCGA	TGTGTCGGGTTCTGAGTAGAG
GZMK	GGGGCTTATATGACTCATGTGTG	GTGGATCAATCAGAACACCTCC
CXCR6	GACTATGGGTTCAGCAGTTTCA	GGCTCTGCAACTTATGGTAGAAG
STAT1	CAGCTTGACTCAAAATTCCTGGA	TGAAGATTACGCTTGCTTTTCCT
β-actin	CACAGAGCCTCGCCTTTGCC	CATGCCGGAGCCGTTGTCG

Figure S1. Risk scores in different lung adenocarcinoma patients.

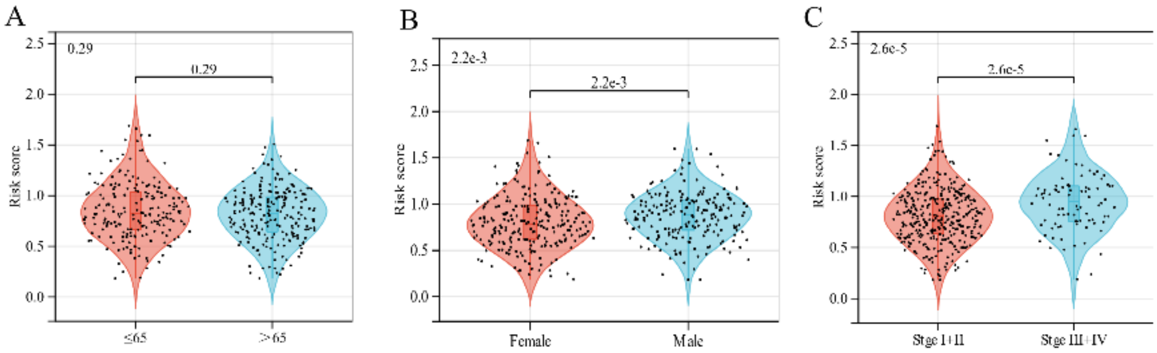

(A) Risk scores between patients ≥65 years old and <65 years old; (B) Risk scores between female and male; (C) Risk scores between patients of pathological stage I and pathological stage II.

Figure S2. Correlation of IARGI risk score with PDL1 expression, tumor mutational load (TMB).

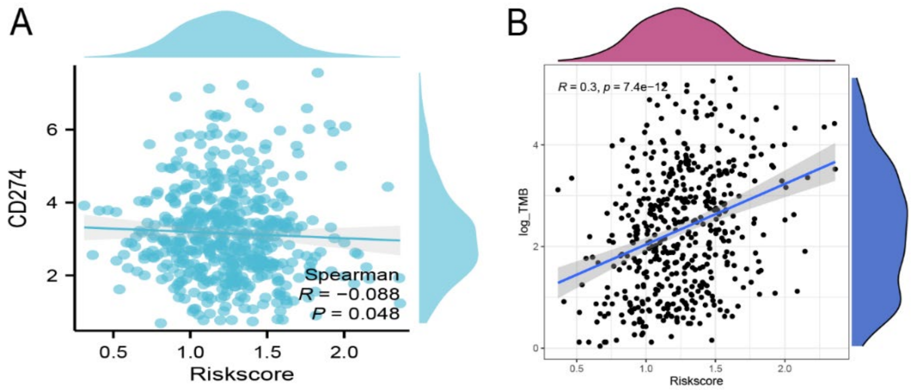

(A) IARGI risk score negatively correlates with PDL1 expression; (B) IARGI risk score positively correlated with TMB.
